# Was it worth it? Older adults’ experiences of participating in a population-based cohort study – a focus group study

**DOI:** 10.1186/s12877-019-1238-4

**Published:** 2019-08-19

**Authors:** Synneve Dahlin-Ivanoff, Therese Rydberg Sterner, Kaj Blennow, Ingmar Skoog, Hanna Falk Erhag

**Affiliations:** 1 Center for Ageing and Health – AGECAP, Gothenburg University, Sahlgrenska Academy, Wallinsgatan 6, SE-431 41 Mölndal, Sweden; 2 Institute of Neuroscience and Physiology, Neuropsychiatric Epidemiology, Sahlgrenska Academy at the University of Gothenburg, Wallinsgatan 6, SE-431 41 Mölndal, Sweden; 3 Clinical Neurochemistry Laboratory, Sahlgrenska University Hospital, Mölndal, Sweden; 4 Institute of Neuroscience and Physiology, Clinical Neurochemistry, The Sahlgrenska Academy at the University of Gothenburg, SE-431 80 Mölndal, Sweden; 5 Institute of Neuroscience and Physiology, Section of Health and Rehabilitation, Sahlgrenska Academy at the University of Gothenburg, P.O. Box 455, SE- 405 30 Gothenburg, Sweden

**Keywords:** Longitudinal population-based cohort study, Research participation, Health research, Focus group method, Older adults, H70

## Abstract

**Background:**

At present, we know relatively little about priorities and problems with topics that older adults experience when completing different examinations in longitudinal population-based studies. To examine these topics, research must be adapted to investigate the meanings, motivations, and interpretations of the individual participants themselves. Therefore, the present study aimed to explore older adults’ motives, understandings and experiences regarding participating in the Gothenburg H70 Birth Cohort Studies (the H-70 study).

**Methods:**

Focus group discussions were used. A total of thirty-eight persons, 19 women and 19 men participated in nine focus groups. A strategic sampling technique was used to ensure that the focus group participants represented the larger population.

**Results:**

The results supported the overall theme: “It was well worth the effort,” which summarized how the participants felt about the population health study. The following specific themes were also identified: an intense event, for the benefit of oneself and others, confidence in health research and the researcher, key decisions about test outcomes and the survey raising questions and providing few answers.

**Conclusions:**

Knowledge of priorities and problems with topics experienced by older adults completing different examinations when participating in longitudinal population-based studies is crucial for research to improve the health and wellbeing of older people. To date, older people’s involvement in population-based cohort studies has largely been as research subjects. This study is a first step toward the participants taking a more active part by allowing them to share their experiences which can be used to improve the research procedures. This requires the participation of older adults in collaboration with the researchers, to ensure the quality of longitudinal studies of older adults. Therefore, our intention when it comes to future research will be to involve older adults—the target group—in the research procedure.

## Background

Longitudinal population-based cohort studies that assess health-related exposure outcomes can provide important knowledge about the needs of older adults [1] and are critical to our understanding of the aging process [2]. One of the difficulties associated with conducting longitudinal population-based cohort studies is attrition (i.e., the loss of study participants), which can occur for several reasons such as death, frailty, withdrawal, or lack of success in re-contacting participants for follow-up examinations. Several studies have identified factors explaining attrition as well as reasons why older adults choose to participate in longitudinal population-based cohort research. Very few have focused on how older adults experience participating in the studies. However, most of these studies have used quantitative methods and focused on describing the characteristics of older participants and non-participants in relation to dropping out, such as educational level, cognitive ability, and marital status [3–6]. Although these studies point toward important factors for attrition and study participation, research focusing on the experience of older persons participating in longitudinal population-based health research is scarce.

Getting the full picture calls for a qualitative approach that explores older adults’ experiences of participation in longitudinal population-based cohort studies, as well as their motives to participate. Qualitative approaches, in contrast to quantitative approaches, do not emphasize the measurement and analysis of cause–effect relationships between variables. Instead, qualitative approaches stress the socially constructed nature of reality and the subject’s perspective, seeking to gain a better understanding of what older people experience when participating in such studies, and why they participate. To our knowledge, only one study conducted by Mein and colleagues [7] has focused on older adults’ experiences of participating in a longitudinal population-based cohort study. Their results showed that, the main motives for and experiences of participation was the personal benefit they perceived, especially the information and care received during medical examinations and the sense of loyalty and membership associated with being part of the study [7].

Exploring the experience of participation among older adults, as well as their motivations to participate, could allow for the identification of appropriate incentives for participation in future studies, ensuring that these studies are acceptable, convenient, and rewarding for the participants [2, 7]. At present, we know relatively little about the priorities and problems with topics experienced by older adults completing different examinations when participating in longitudinal population-based studies. To examine these topics, research must be adapted to investigate the meanings, motivations, and interpretations of the individual participants themselves [8]. Therefore, the present study aimed to explore older adults’ motives, understandings and experiences regarding participating in the Gothenburg H70 Birth Cohort Studies (the H-70 study) based on the following research questions;

What made them accept participation in the H70 study?

How did they experience the examinations?

How did they experience the information concerning the examinations?

How did they experience the questions in the examinations?

## Methods

### Design

Focus-group methodology differs from much qualitative research in that it is based on a collective understanding of participants’ views [9]. When planning this study, we discussed whether to use focus groups or individual interviews. The collective nature of focus groups can, according to the literature [9], empower the participants, and focus groups are especially useful for engaging people with limited power and influence, such as, for example, older adults. The method utilizes the interaction among research participants to generate data that are used in the study [9–11]. The shared experience can be a powerful tool for expressing itself both negatively and positively, which the individual interview does not provide the same conditions for. This was important reasons for the choice of the focus group method. The Regional Ethics Committee at the University of Gothenburg approved the study (869–13, T915–14).

### The H70 study

This study is part of the Gothenburg H70 Birth Cohort Studies. The overarching aim of the H70 studies is to examine the impact of mental, somatic and social health on the functional ability and well-being of individuals aged 70 years and above, taking into account the complex interactions with age, sex, gender, socioeconomic gradients, environmental exposures, psychosocial, neurobiological, and genetic factors. The research gained from the H70 studies has clinical relevance in relation to prevention, early diagnosis, clinical course, experience of illness, understanding pathogenesis and prognosis. Results will increase our understanding of ageing and inform service development, which may lead to enhanced quality of care for older persons. The first H70 study started in 1971. New birth cohorts of 70-year-olds have been added over time. To date, the ongoing H70 study comprises six birth cohorts. Longitudinal follow-up examinations have been conducted or are planned at ages 75, 79, 85, 88, 90, and 95+ for most cohorts. This paper is based on the study procedures for the baseline examination of the Birth cohort 1944, conducted in 2014–16. This was the largest and most comprehensive H70 study conducted so far (*n* = 1203). As in previous examinations, data collection serves as a basis for future longitudinal follow-up examinations [12].

All participants were invited to participate in general examinations and additional examinations. The general examination comprised blood sampling, genetics and family history, psychiatric examination, clinical cognitive examination, psychometric cognitive examination, general health interview, medications, physical examination, ECG, basic body composition, lung function examinations, spirometry, PEF, audiological examination, ophthalmic examination, functional ability and disability, physical fitness and physical activity, social factors and self-rating questionnaires. After the general examination, all study participants were asked to take part in additional examinations at a later date: close informant interview, dietary examination, body composition examination, computerized tomography (CT) scan and pre-symptomatic testing; magnetic resonance imaging (MRI) of the brain, and cerebrospinal fluid collection by lumbar puncture (LP). Subsamples were invited to extended audiological, and ophthalmological examinations, and qualitative studies. The participants were able to choose not to take part in the examinations. The full set of added H70 study examinations required approximately 14 h to complete, spread across several days. For a more meticulous information see study protocol [12].

### Participants

The sample was derived from the population based H70 Study (baseline examination for birth cohort 1944). Participants who had taken part in H70 examinations during May to December 2014 were eligible for participation in the focus groups. In this study, we used a purposeful sampling strategy to ensure that the focus group participants represented the larger population of H70. A total of thirty-eight persons participated in nine focus groups (3 to 6 participants/ group, median 5) from February to September 2015. To create focus groups both homogeneity and heterogeneity are important when selecting participants. Homogeneity, is about sharing similar experiences for creating discussion. The focus groups participants shared the experiences of being 70-year-old and having participated in the H70 study. Heterogeneity, is needed to cover diversity within the chosen target group. Diversity in the focus groups was considered in terms of differences in sex, marital status, profession and education. Heterogeneity was also considered when it comes to participation –nonparticipation in the LP examination. This because the participation rate was high for all examinations in the H70 study, except for the LP (27%). Therefore, the focus groups were put together so that they consisted of both participants as well as nonparticipants in the LP procedures in order to represent the larger population of H70.

### Procedure

Five individual pilot interviews with open-ended questions were conducted to test the feasibility of the key questions and to ensure that the sensitive topics were suitable for focus groups. The eligible participants were contacted by post followed by a telephone call. Written and verbal information concerning the study’s aim and procedure was provided. It was stressed that participation in the focus group discussions was voluntary and that all information would be handled confidentially.

The focus group discussions were conducted in a university conference room. Each focus group lasted 2 hours or less. The sessions began with the moderator informing the participants about the study, its purpose, and the structure of the focus group, making clear that we wanted to learn from the participants and that they were the experts. The participants introduced themselves and told the other group members a little about themselves. The moderator then introduced the discussion topic and the participants were encouraged to discuss the topic openly. The discussions were based on the research questions. The moderator’s task was to pose questions to deepen the discussion and ensure that all participants were given a chance to speak, identifying common elements in the discussions and posing general questions followed by more specific questions.

### Data analysis

The analysis was based on a method developed by Kreuger and Casey [13]. All sessions were audio-recorded and transcribed verbatim. It was important to keep the raw data in view long enough to understand the meaning of the material. The analysis was conducted in Swedish as far as possible. To become familiar with and gain understanding of the content of the material in its context, the first step in the analysis was listening to the audio recordings several times, guided by the purpose of the study. At this stage, the working material was still in the form of raw data. The transcript of each group discussion was then read carefully and independently by each of the authors to get an overall sense of the data. Next, sections relevant to the research topic were identified and sorted into different themes. Focus groups generate a large amount of data, and it can be difficult to get an overview. The aim of the study guided our comprehension of the contextual meaning of the material. Categories that emerged from our review of the raw data were defined, and descriptive statements synthesizing, abstracting, and conceptualizing the data were constructed. The last step was to summarize the categorized raw data, combined with an interpretative step aiming to provide understanding.

The data analysis process was iterative; that is, each step was initially conducted by the first author separately and was then discussed by all of the authors. The level of coherence was very high, although the authors sometimes used different words to describe the same results. Any disagreement among the authors was resolved by discussion.

## Results

### It was well worth the effort

**Fig. 1:**
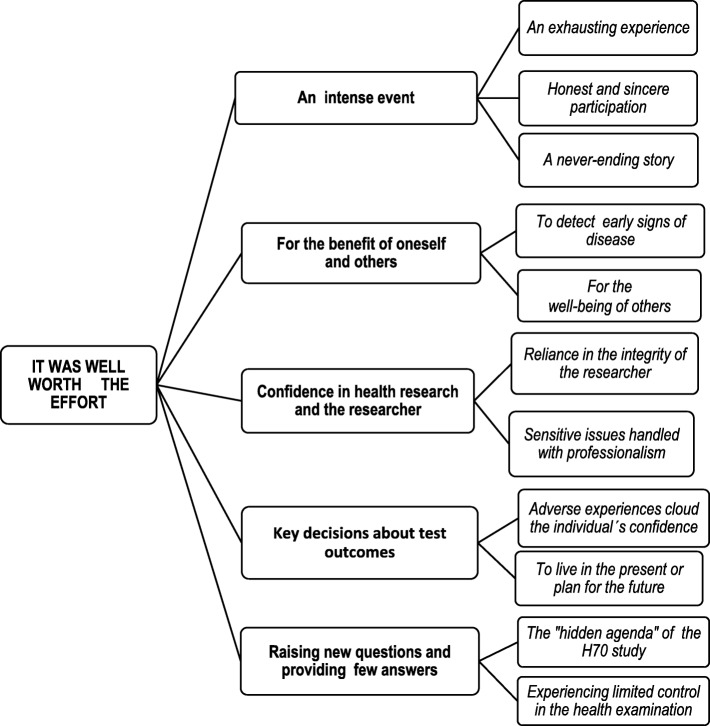
Results by overall theme, themes and sub-themes

The results tended toward unanimous support for the overall theme: “It was well worth the effort,” which summarizes how the participants felt about the health population study. Despite expressing mixed views concerning various aspects of participation, the study was described as being worthwhile, overall, in accordance with the following specific themes: an intense event, for the benefit of oneself and others, confidence in health research and the researcher, key decisions about test outcomes and the survey raising questions and providing few answers (see Fig. 1).

### An intense event

The participants experienced the H70 study as an intense event—both exciting and challenging. If they were given the opportunity to take part in a similar study again, they would do so. The sub-themes that emerged from this theme included “an exhausting experience”, “honest and sincere participation” and “a never-ending story.”

### Citation focus group five (three women)

*I: But you weren't nervous about this whole day of examination?*

*P1. No, no*

*P2. I thought it was exciting. Yes it was fun; you didn't know at all what it was. No. They put a couple of stuff on the table.*

*P3. Yes yes sure. Thought it was a little tough.*

*P2. There were a lot of questions*

*I: Yes*

*P1. And it went pretty fast. Yes, I do not know if we were in a hurry, everything was just about done before lunch, all these questions and you had to look at pictures. Yes at last I became almost whimsical. I thought so; I thought it was very much there.*

*I: It was an exhausting day*

*P2. Yes, you were completely empty in some way I thought then. And then when we went home I thought but oh what have I been through.*

*. Yes [all]*

#### An exhausting experience

The H70 study was found to be an exhausting experience, which had not been anticipated by the participants. It included many questions, tests, and assessments, which were carried out at a fast pace. The participants experienced tension and fear because they needed to perform unpleasant tests such as the LP or MRI, and they were also expected to provide spontaneous responses, which required extra effort. Participation was experienced as tiresome; by the end of the study, the participants felt mentally exhausted and unsure whether they had understood the information accurately. However, despite these views and the intensive nature of the event, the participants experienced the study as exciting.

#### Honest and sincere participation

As the H70 study was an intensive event the participants were concerned about whether they had reported their feelings and experiences correctly and unsecure about whether they had actually responded honestly to the questions. The participants expressed a desire to perform as honestly as possible, to do the “right thing,” and to be as honest as they could be in providing responses and undergoing examinations and assessments.

#### A never-ending story

The participants felt that the H70 study was carried out over a long period, and new invitations to complete additional tests, assessments, and questionnaires were sent out continually. The participants reported being unaware of the extent of the study when they agreed to take part. The participants discussed whether the researchers’ strategy of not disclosing the extent of the study to the participants affected the number of assessments they agreed to complete. They also discussed whether their lack of knowledge of the extent of the study was the responsibility of those conducting the H70 study, which could be interpreted as withholding the truth from the outset in an attempt to increase study participation. However, the discussions also showed that the demanding experience was considered worthwhile because of its benefits.

### For the benefit of oneself and others

Participants agreed to take part in the H70 study based on thinking that the study could be of use to themselves, as well as to others. Participation in the H70 study was seen as a major advantage because participants believed that they experienced inequity in health care treatment because of their age.

The sub-themes that emerged from this theme included “to detect early signs of disease” and “for the wellbeing of others.”

### Citation focus group eight (two women and one men)

*I: Yes was it a contribution to the research or was it for your own part or how did you feel?*

*P1. To research…. I thought it was good if I could contribute a bit with me and my way of being and my way of life and so, and get my values ​​then*

*P2. Yes it felt so well, that someone gets the benefit out of it, but I also wanted to have something out of it for me personally. I just mean to run all the values ​​into a bank, read statistics and the like, it is of course good*

*P3. I didn't think like that...*

*P1. No but*

*P3.. I didn't think I wanted to benefit from it*

*P. I thought so*

*P2. I wanted (to benefit), I felt it*

*P1. I also wanted that, I thought it was very good to get sort of a check; I really thought was really great*

#### To detect early signs of disease

This sub-theme concerns the participants’ actions to improve their own health. The driving factor for study participation was the opportunity “to be caught” before anything unhealthy occurred that would result in the participants ending up in the healthcare system. They consented to participate in the research because they felt that the healthcare system did not function in a satisfactory manner. They felt that they would never receive such an extensive examination with their own doctors. If they were indeed sick, they would receive follow-up care and be referred to a recommended doctor through the study. Additionally, it was considered a positive result if they were deemed healthy, and they would receive a certificate to prove that fact.

#### For the wellbeing of others

Contributing to research that is beneficial to others was identified as an important reason for study participation. Participants hoped that their research participation would influence both the conditions of the aging process and the opportunities and possibilities for leading a healthy and fulfilling life. Participants also saw this opportunity as beneficial for their children, grandchildren, and future generations. Thus, the future was also considered important. Participants saw their own participation in the H70 study as having a positive influence on the future health of their loved ones. This ambition was identified as an additional factor encouraging agreement to take part in the study and making the study a worthwhile cause in which to participate.

### Confidence in health research and in the researchers

In addition to the approach of the study and how it was experienced, participants also discussed how the research that emanated from the H70 study had a purpose. They argued that the study and its questions were necessarily in-depth for research purposes. The participants considered how they were addressed and the questions they were asked to be important in developing trust in the researcher and the research. The sub-themes that emerged from this theme included “reliance on the integrity of the researcher” and “sensitive issues handled with professionalism.”

#### Reliance on the integrity of the researcher

Confidence in the researcher depended on trust and on the relationships with the research staff such as professionals, researchers, research nurses and research assistants conducting the examinations, tests, and assessments. When the participants experienced a sense of safety and felt trust and confidence in the researcher, it was not perceived as difficult to be as open and truthful as possible or to feel safe and secure when completing the examinations and tests. This was particularly true when the participants encountered personnel who were able to provide information not only in an understandable manner, but also in a non-discriminatory manner. The participants described the research staff member as crucial in their decisions about whether to agree to certain tests and whether to answer the questions truthfully. Feeling safe with and confident in the research staff member meant that participation did not feel difficult or unpleasant.

#### Sensitive issues handled with professionalism

The participants claimed that sensitive issues did not remain at their original level of sensitivity if they were handled with professionalism. Certain assessment questions gave rise to uncomfortable emotions or made the participants aware of issues that they experienced as uncomfortable or unpleasant. Questions arose that could have been perceived as very discriminatory and/or offensive, such as very basic questions that were not perceived as relevant to a 70-year-old. The participants felt that the questions were more relevant to an older age group because they regarded themselves as being too healthy to answer these questions. Some questions were also regarded as heteronormative. The person asking the questions and how they dealt with the participant was very important; that person was seen to have a major responsibility when dealing with potentially sensitive subjects. The person asking the questions carried a major responsibility if a topic or issue was difficult to respond to or if the participant did not understand the question or felt worried or fearful about undergoing an examination or test. Even so, participants mentioned feeling that they had to answer the questions because they were being asked for research purposes.

### Citations focus group 7 (three women and three men)

*I: Do you think that overall it has been a good interaction with the staff, is there something you would like…?*

*P1. No, since it has been a good response*

*P2. Incredibly good, I think, very comfortable and nice*

*P3. But if you go into a thing like this, you have to have confidence and you have to trust people, otherwise it is like no idea*

*P1. Otherwise, you probably won't participate throughout this study*

*P4. You assume that too. And maybe we are all here around the table people who want to contribute, I don't know but I get a feeling of it anyway*

*I: So it is important with confidence then, trust?*

*P5. Yes*

*I: And the staff has transpired that, that's what you really say?*

*P6. Well, and I assume that they also have.*

*P2. Felt very well cared for*

*P3. Well they were very professional, pretty much everyone*

*P5 .Yes, I do think that*

### Key decisions about test outcomes

This theme concerns the participants’ willingness to take part in tests such as the LP and MRI, as well as their experiences of these tests. LP and MRI were those of the various examinations that stood out as a very unpleasant experience for the participants. The participants asked themselves two questions: “Do I want to do the test?” and “Do I want to know the result?” The sub-themes that emerged from this theme included “adverse experiences cloud one’s confidence” and “to live in the present or plan for the future.”

#### Adverse experiences cloud one’s confidence

Earlier personal experiences and the experiences of others had an impact on participants’ responses to the question “Do I want to do the test?”. Stories heard from friends or relatives describing the LP and MRI as unpleasant experiences that could do more harm than good, affected the participants’ confidence levels and became a reason for saying “no.” In contrast, participants felt that the H70 study was a positive event, and this was a reason for responding “yes.” Having a needle inserted into one’s back or feeling trapped inside an MRI machine could be experienced as more or less or unpleasant, based on possible earlier experiences. Participants expected the LP to be painful because they had heard and read about it previously. The information they received as part of the study stated that the experience should not be painful, but the participants reported experiencing more pain than this information had led them to believe they would feel. Participants were also afraid of side effects: They had heard stories about people who had undergone an LP many years ago and suffered permanent injury as a result. In terms of MRI, the examination was experienced as a very unpleasant test that was noisy, with “knocking and banging.” The participants said that these noises were penetrating, and steel fixtures were uncomfortably located just above their faces. The participants were afraid that they would be unable to handle this unpleasant experience. Furthermore, the advice to listen to music through headphones was not always useful because the noise of the procedure drowned out the music.

#### To live in the present or plan for the future

There were two schools of thought regarding whether to find out the test results: to live in the present or to plan for the future. These approaches could be affected by whether a cure existed in the form of anti-retroviral drugs or whether there was a hereditary predisposition for the illness. Some people chose to live in the “here and now,” implying that they took each day as it comes; these people would respond “no” to learning their test results, because this knowledge would impact their quality of life. The suspicion that they may have the disease would have always been there, affecting their everyday lives. The knowledge that they were at risk would mean that they would search for symptoms and signs, either in themselves or in others who were at risk. However, some people wanted to know their test results so that they could plan for the future. These people did not want to become a burden on their families, affecting those closest to them. This implies planning for what may happen, for example by making preparations such as acquiring a new place to live or letting people know they belonged to a certain risk group.

### Citations focus group 7 (two women and one men)

*I: But you didn't want to find out*

*P1. No I didn't want to*

*I: But you did it anyway*

*P1. Yes, because I said this, if there is anything you see that can do something about, then I want to know, but this with Alzheimer's and dementia that cannot, I do not want to know*

*P2. We sweep under the carpet*

*P1. I feel like this that if I could find out, or would I find out then I can make some preparations*

*P3. What can you do?*

*P2. Yeah, but maybe I can*

*P1. Moving over money ...*

*P2. Among other things, or try to eventually get me some other accommodation and tell people to, yes prepare so that, yes, no I would love to know*

*P1. I would also, yes absolutely*

*P3. Yes no I asked my daughter what she thought, because it will be she who is affected, not me*

*P2. Yes, but it is good for her to know*

*P3. No she didn't want to know*

*P2. But why does mom forget all the time and why does she keep on asking the same things*

*P3. No, we'll take it when it's time in that case, she said*

### Raising new questions and providing few answers

The study raised additional questions because of lack of understanding, and the participants remained uncertain as to whether they received all of the information. The participants demonstrated a strong desire to complete examinations with the research staff they met, where they were able to undergo an overall assessment and reach a conclusion. Despite the participants’ points of view, their participation in the H70 study felt it worthwhile because of its benefits. The sub-themes emerging from this theme included “the hidden agenda of the study” and “experiencing limited control in the examination.”

#### The “hidden agenda” of the health study

Participants expressed suspicion about whether they really received all of the information, as well as whether they received truthful information. Many questions in the interviews or in the self-rating questionnaires were highlighted as sounding similar, being asked in different ways but with the same purpose. The questions were first biased in one direction and then reappeared later biased in another way. Participants discussed the purpose of this repetition: Were the repetitions intended to fail for the participant, or what did the questions actually intend to test? The participants believed the study to be more advanced, with a “hidden agenda” lurking somewhere.

#### Experiencing limited control in the health examination

The participants felt that they did not always know which examinations had been carried out. They could not understand all that had been said, and they desired more information to understand. The participants expressed that they did not completely understand the intention and purpose of some of the tests, which raised several unanswered questions about the different examinations. The participants expected to get more explanations and answers about what they had experienced as part of the study, especially if any deviations existed, and they also expected to receive advice and recommendations. They sometimes received advice, but sometimes they did not, despite their understanding that they would receive that information. They could not always understand whether a test result was good or bad. The participants said that receiving some form of feedback after some of the examinations would be beneficial, but not all of the participants received this feedback.

## Discussion

The H70 study was experienced on the one hand as intense, exhausting, never-ending, and on the other hand as interesting, rewarding, challenging and beneficial - both for oneself and others. So, even though the participants have some critical views on the H-70 study as a whole, the results showed that, despite these critical views, the participants trust the intentions of the research and the researcher. Tensions can arise between trust for the research and mistrust such as for example the participants believed the study to be more advanced, with a “hidden agenda” lurking somewhere or whether they received truthful information. This could be reflected in the view of Wright’s [14] definition of trust/mistrust. A trusted researcher recognizes the value of the trust that the participant vests in them and uses this to rationally determine how to act. Vice versa, distrust is defined as the participants’ belief that his / her needs are inferior to the researcher, as shown by conditions such as the storage of important information, risks that consider benefits or data that harm persons or communities. However, despite signs of mistrust the participants found the study was well worth the effort.

Trusting the researcher and the research is considered an essential prerequisite for involvement in health research [15, 16]. In their research, McDonald and colleagues [16] found that trust is a dynamic concept involving building a relationship and interacting in respectful ways, but this trust can easily be broken. Another example of complexity is Morris and Balmer [17] research on participation in a biomedical research experiment. The research participants, in order to establish a relationship, in which they can feel socially comfortable and valued, moved through multiple roles through a unfamiliar social territory. The researcher [17] highlight that the negotiation about the relationship can have both positive and negative outcomes. A positive outcome is a mutually agreed relationship important for both parties, and for the conduct of the research. In our research, the participants described during all examinations, that the relationship were of the utmost importance for the quality of the research but also for research outcomes.

People participated in the H70 study for the benefit of themselves. They expressed that their participation was an opportunity to detect early signs of disease, because the health examinations in the study were more extensive than those normally offered by their doctors. Similar results were found by Mein and colleagues [7], who reported that the main motives for older participants to take part in longitudinal population-based health research studies were the medical examinations and the personal benefits these entailed. Previous health research studies using diverse research designs and studying various age groups have found that research participation is a way of gaining access to health care that is otherwise unavailable or difficult to get [15, 18].

The participants reported that taking part in the H70 study was motivated by a major advantage as they experienced unequal access to health care because of their advanced age. Research into age discrimination has shown differences in health care services offered by age, with elderly people receiving less favorable treatment [19]. Townsend and Cox [18] have stressed that access to health care services through research participation points to systemic inequities in health care. The desire for participation in research, raise the question of whether those with the fewest health care options, take considerably greater risks, that is, uncertainty about direct therapeutic benefit and research boundaries compared with the rest of the population. However, it is worth considering whether it is those older adults who decline to participate who take more risks, because they are probably frail and have fewer health care options. Townsend and Cox [18] have argued that greater attention must be given to ascertaining the consequences of seeking health access through the “back door” of research participation. If research is a way to gain access to treatment, we must be concerned about the context in which decisions on research participation are made and what consequences this will have for research participation. This is important as Swedish health care has undergone dramatic changes during the last decades, with decreased number of hospital beds and shorter hospital stays [20], especially evident concerning geriatric hospital care [21]. Despite health care in Sweden being one of the best worldwide, many older people do not receive appropriate health care.

Another motivation for participating was a concern for the wellbeing of others. Several studies have shown that altruism is a motivator for participation in research [22, 23]. In the H70 study, the participants, in addition to participating for the sake of the research itself, saw this opportunity as beneficial to the future health of their children, grandchildren, and future generations. This finding contrasts with the longitudinal population-based health research study by Mein et al. [7], which found that the research staff credited the participants with altruistic motives but that the older people did not report this themselves. Similar to the present study, other past work [23] has found that benefits for themselves and for others combined to motivate people to participate.

LP and MRI were those of the examinations that stood out as a very unpleasant experience for the participants. The participants asked themselves two questions: “Do I want to do the test?” and “Do I want to know the result?” Townsend and Cox [18] have claimed that research participants seem to be well aware of the risks they face, the uncertainty of the therapeutic benefits, and the limits of the research. However, according to Hallowell and colleagues [23], motivation to participate involves much more than just weighing the risks vs. benefits: Participants perceive themselves as positioned in a complex and dynamic network of relationships where their actions are linked to others in the past and the future, and participants’ motives are influenced by a combination of reasons such as, for example, personal gain; benefits to their children, grandchildren, and others; and the influence on the wider community [23]. In our study learning one’s LP or MRI test results was a conscious decision and the participants weighed the risks to themselves and how it would affect their relatives and their wider social context.

The participants experienced limited control over the examination, not understanding all of the information that had been given and not completely grasping the intention and purpose of the various tests. This resulted in several unanswered questions. Health literacy is the ability to handle information, including the comprehension of verbal and written health information [24] . Low health-literacy skills can affect all age groups [25] but when we age, people become more vulnerable to inadequate health literacy [26]. The H70 study is a complex environment that poses major challenges for participants’ ability to understand and use health information.

It is important to build a health-literate health research organization, implement educational tools tailored to professionals likely to interact with persons with poor health literacy. It is also important to implement interventions to improve the organizational ability to identify and meet the knowledge needs of people with marginal health literacy skills [27, 28].

The present study has also shown the importance of good communication in all examinations. Kaphingst and colleagues [29] have emphasized not underestimating the importance of effective, respectful engagement from staff members, in addition to each person’s health-literacy skills. Mein and colleagues [7] found that participants appreciated the interaction with staff members, describing them as “friendly,” “polite,” and “professional,” and noting their efforts to make the medical examination more “personal”. They have reported that older adults’ participation in a longitudinal population-based study was experienced as burdensome [7]. Similar to our study, their results showed that the participants strongly disliked completing the comprehensive questionnaire. Mein and colleagues [7] also reported that the participants commented on the type of questions and the repetition of questions. In our study, the questions were considered more or less sensitive by the participants such as the participants perceived certain questions as ageist attributing certain characteristics to older adults because of their age. Other questions implied age-related functional limitations that were perceived as not relevant to a 70-year-old person, but rather to individuals in older age groups with more health problems. In addition, some questions were perceived as heteronormative, suggesting that heterosexuality is the only sexual orientation, which the participants also regarded as old-fashioned and not in line with contemporary societal views on sexuality. Our results showed that questions about sensitive issues were not perceived as being as sensitive if handled with professionalism. If the participants felt safe and confident, they did not find it difficult to be open and truthful when answering sensitive questions. The research staff and communication play critical roles in the development and operation of a health-literate organization, that is, the ability of the organization to establish friendly and comfortable relationships the participants [28].

Another issue related to limited control over the examination is that the participants felt that some of the research staff gave answers and advice, whereas others did not. It is possible that this is because of the dominant (within classical medical research) positivistic scientific view, which is based on objectivity, meaning that the same questions and information are given to everyone. This has been discussed by Mein and colleagues [7], who pointed out that nurses/researchers are not “allowed” to give medical or health advice to research participants. This creates a conflict between the positivist scientific view and person centeredness, that is, an encounter that is achieved through a dynamic relationship on equal terms between the older person and the researcher [27], as opposed to the standard questions (the same question to all). This may affect the quality of the H70 study, as the standard questions may not always be so clear that they are understood by the participants. In addition to the fact that there always ought to be an active reflection going on within the H70 study team on which scientific view the study represents and how it is implemented, there is also recognition that the participants need to be involved in the research. This could mean, among other things, improving information and questionnaires in collaboration with the research participants to ensure the quality of the H70 study [28].

### Limitations

Careful group composition is important in focus groups to prevent strong uniformity in the groups [13]. Therefore, great efforts were made to assemble participants with common experiences that varied in nature, taking into account both heterogeneity and homogeneity. A carefully considered group composition can also avoid a situation where the moderator interviews the group, rather than creating interaction between the group participants. What united our participants (homogeneity) was their common experience of participating in the H70 study. Previous research has shown that being grouped with others with the same experiences, being able to discuss things with people who understand, and knowing that you are not the only one with a particular experience create a feeling of sharing [9]. The participants in this study seemed to appreciate the opportunity to take part in the focus groups, resulting in fruitful discussions in which the participants shared their views—both positive and negative. Negative views have been found to be more easily expressed in the presence of other participants having something in common [9, 13]. It was stressed that the participants were experts, and they were able to express their views on a relevant subject; this gave them a strong voice, which they appreciated.

Each of the focus groups in the present study had a limited number of participants. Most recommendations in the literature are for larger focus groups, with up to 12 participants [10]. In this study, we planned for six participants in each group, but the actual numbers were one group with six participants, four groups with five participants, and four groups with three participants. Small groups of three to six participants have been shown to be very dynamic, and the outcome of the discussion depends more on the involvement of the participants than on the number of participants [9, 10].

The credibility of focus group results increases if several focus groups are implemented. In this study, thirty-eight persons participated in nine focus groups, which can be seen as a good number for high credibility. Another issue that could be discussed is if bringing together participants in focus groups would affect the responses to questions in the next wave, so that the cohort no longer follows a “natural” course. Considering the number of participants that participated in the focus groups (*n* = 38) compared to the number of persons participating in the H 70 study (*n* = 1203), the possible impact on the “natural course” is negligible compared to the benefits to the main study.

One criticism of the focus group method is that it could be difficult to get participants to address sensitive topics and that individual interviews are therefore preferable. It certainly may be true that there can be a high level of trust in an individual interview, resulting in in-depth data that may not be achievable in group discussions. However, in group discussions, the group identity and recognition between the participants can make them share things that they would be unable or unwilling to recount in individual interviews. The collective nature of focus groups can, according to the literature [9], empower the participants, and focus groups are especially useful for engaging people with limited power and influence, such as, for example, older adults. Feeling a sense of fellowship with others in similar situations may encourage research participants to express things that would not be discussed otherwise [29]. The awareness of sharing similar experiences can make participants realize that their views are legitimate and valid [9, 10, 13] which was the case in the present study.

## Conclusion

The Gothenburg H70 Birth Cohort Study was well worth the effort. The participants trusted the intentions of the research, even though they were critical of the examinations, the questions and the information. If we want adequate insight into the subjective experience of participating in health research, qualitative research is one way for participants to take part in or contribute to the development of better research procedures [30, 31]. Another important initiative of interactive dynamic research model with the participants is the All of Us Research Program [32]. Their main aim is to build a research model for exploration of biological, social, environmental health determinants of health and disease. Including participants as partners in the development and implementation of the research is a central principle.

Knowledge of priorities and problems with topics experienced by older adults completing different examinations when participating in longitudinal population-based studies is crucial for research to improve the health and wellbeing of older people. The study has provided us with knowledge concerning a number of problems what we have to consider in future examinations. To date, older people’s involvement in population-based cohort studies has been more as research subjects, and this study is a first step toward the participants taking a more active part by allowing them to share their experiences which can be used to improve the study procedures. To gain this we need to create a supportive environment based on competence and well-trained staff with access to the educational tools to secure the quality of the results the H70 study. It also includes development of tools needed to meet the needs of people with lower health literacy. This requires the participation of older adults in collaboration with the researchers, to ensure the quality of longitudinal studies of older adults. Therefore, our intention when it comes to future research will be to involve older adults—the target group—in all stages of the research process.

## Data Availability

This is a qualitative study and the data generated in the study are not available in line with information provided to the participants in the informed consent and all attempts would be made to maintain confidentiality.

## Abbreviations

CT: Computerized tomography
LP: Lumbar puncture
MRI: Magnetic resonance imaging
The H-70 study: The Gothenburg H70 Birth Cohort Studies
